# CSF protein biomarkers are associated with atrophy and symptom severity in genetic FTD: a GENFI study

**DOI:** 10.1007/s00702-026-03118-y

**Published:** 2026-02-19

**Authors:** Abbe Ullgren, Melissa Taheri Rydell, Sofia Bergström, Linn Öijerstedt, Jennie Olofsson, Arabella Bouzigues, Lucy L. Russell, Phoebe H. Foster, Eve Ferry-Bolder, John C. van Swieten, Lize C. Jiskoot, Harro Seelaar, Raquel Sanchez-Valle, Robert Laforce, Daniela Galimberti, Rik Vandenberghe, Alexander Gerhard, Simon Ducharme, Christopher R. Butler, Elizabeth Finger, Maria Carmela Tartaglia, Mario Masellis, James B. Rowe, Matthis Synofzik, Fermin Moreno, Barbara Borroni, Jonathan D. Rohrer, Peter Nilsson, Elena Rodriguez-Vieitez, Anna Månberg, Eric Westman, Caroline Graff, The Genetic Frontotemporal Dementia Initiative (GENFI)

**Affiliations:** 1 Department of Neurobiology, Care Sciences and Society, Center for Alzheimer Research, Division of Neurogeriatrics, Karolinska Institutet, Solna, Sweden; 2 Institute of Clinical Medicine – Neurology, University of Eastern Finland, Kuopio, Finland; 3 Unit for Hereditary Dementias, Theme Inflammation and Aging, Karolinska University Hospital, Solna, Sweden; 4 Division of Affinity Proteomics, Department of Protein Science, KTH Royal Institute of Technology, SciLifeLab, Stockholm, Sweden; 5 Dementia Research Centre, Department of Neurodegenerative Disease, UCL Queen Square Institute of Neurology, London, UK; 6 Department of Neurology, Erasmus Medical Centre, Rotterdam, Netherlands; 7 Alzheimer’s disease and Other Cognitive Disorders Unit, Neurology Service, Institut d’Investigacións Biomèdiques August Pi I Sunyer, Hospital Clínic, University of Barcelona, Barcelona, Spain; 8 Département des Sciences Neurologiques, Faculté de Médecine, Clinique Interdisciplinaire de Mémoire, CHU de Québec, Université Laval, Quebec, QC Canada; 9 Ospedale Maggiore Policlinico, Fondazione IRCCS Ca’ Granda, Milan, Italy; 10 Dept. of Biomedical, Surgical and Dental Sciences, University of Milan, Milan, Italy; 11 Laboratory for Cognitive Neurology, Leuven Brain Institute, Department of Neurosciences, KU Leuven, Leuven, Belgium; 12 Neurology Service, University Hospitals Leuven, Leuven, Belgium; 13 Division of Neuroscience and Experimental Psychology, Wolfson Molecular Imaging Centre, University of Manchester, Manchester, UK; 14 Departments of Geriatric Medicine and Nuclear Medicine, University of Duisburg-Essen, Duisburg, Germany; 15 Department of Psychiatry, Douglas Mental Health University Institute, McGill University, Montreal, Québec Canada; 16 McConnell Brain Imaging Centre, Montreal Neurological Institute, McGill University, Montreal, Québec Canada; 17 Nuffield Department of Clinical Neurosciences, Medical Sciences Division, University of Oxford, Oxford, UK; 18 Department of Brain Sciences, Imperial College London, London, UK; 19 Department of Clinical Neurological Sciences, University of Western Ontario, London, ON Canada; 20 Tanz Centre for Research in Neurodegenerative Diseases, University of Toronto, Toronto, ON Canada; 21 Sunnybrook Health Sciences Centre, Sunnybrook Research Institute, University of Toronto, Toronto, Canada; 22 Department of Clinical Neurosciences, Cambridge University Hospitals NHS Trust, University of Cambridge, Cambridge, UK; 23 Department of Neurodegenerative Diseases, Hertie-Institute for Clinical Brain Research and Center of Neurology, University of Tübingen, Tübingen, Germany; 24 Center for Neurodegenerative Diseases (DZNE), Tübingen, Germany; 25 Cognitive Disorders Unit, Department of Neurology, Hospital Universitario de Donostia, San Sebastian, Spain; 26 Neurosciences Area, Group of Neurodegenerative Diseases, Biogipuzkoa Health Research Institute, San Sebastian, Spain; 27 Department of Clinical and Experimental Sciences, University of Brescia, Brescia, Italy; 28 Molecular Markers Laboratory, IRCCS Istituto Centro San Giovanni di Dio Fatebenefratelli, Brescia, Italy; 29 Department of Neurobiology, Care Sciences and Society, Division of Clinical Geriatrics, Karolinska Institutet, Huddinge, Sweden

**Keywords:** Frontotemporal dementia, CSF biomarkers, Neuroimaging, C9orf72, GRN, MAPT, Neurodegeneration

## Abstract

Over the past few years, several fluid biomarker candidates have been proposed for frontotemporal dementia (FTD). We have previously identified CSF proteins that could separate individuals with genetic FTD from controls. However, it is unknown whether alterations in these CSF protein levels are associated with neurodegenerative processes. The aim of this study was to explore how these CSF biomarker candidates correlate with symptom severity as well as cortical and subcortical atrophy. The levels of fourteen proteins were measured in CSF from 202 individuals, 131 mutation carriers with mutations in *C9orf72*, *GRN*, or *MAPT*, and 71 controls, in a cross-sectional subset from the GENFI cohort. The association between the levels of these proteins and CDR plus NACC FTLD-NM sum-of-boxes, cortical thickness, and subcortical volumes were estimated in the mutation carriers. Elevated CSF levels of five out of fourteen proteins were associated with an increased CDR score in the mutation carriers. Additionally, elevated levels of three of these proteins, NEFM, PTPRN2 and SERPINA3, were associated with reduced cortical thickness and/or subcortical volume among all mutation carriers. Some mutation-specific associations were also observed, with SPP1 and CTSS being associated with CDR and atrophy only in *MAPT* mutation carriers, while NPTX2 was specific for *GRN* mutation carriers. As indicated by the association to brain atrophy, the proposed fluid biomarker candidates continue to show promise and additional studies will further elucidate their relationship to cortical atrophy in genetic FTD, and their potential as biomarkers for diagnosis, prognosis, and disease staging.

**Supplementary Information:**

The online version contains supplementary material available at https://doi.org/10.1007/s00702-026-03118-y.

## Background

Frontotemporal dementia (FTD) is a heterogeneous syndrome with a wide range of symptoms such as loss of inhibition and social cognition, language impairment and motor dysfunction. The underlying neuropathology is also variable with different protein aggregates such as tau, TDP-43 and FUS/FET. Furthermore, there is genetic heterogeneity as indicated by the known disease causing mutations in progranulin (*GRN*), microtubule associated protein tau (*MAPT*) and chromosome 9 open reading frame 72 (*C9orf72*) as well as several other genes [1].This complexity presents a challenge when it comes to diagnosing patients with FTD, and finding biomarkers that can aid in the diagnosis or serve as markers of underlying pathology and disease severity would be of immense clinical utility.

Previous studies have identified several promising imaging and protein biomarker candidates. For example, presymptomatic regional gray and white matter abnormalities measured via magnetic resonance imaging (MRI) are associated with future clinical decline in genetic FTD [2, 3]. Elevated plasma levels of the protein neurofilament light chain (NEFL) can predict phenoconversion in presymptomatic individuals and correlate with disease severity [4]. However, increased NEFL levels are not exclusive to FTD [5, 6]. Neuronal pentraxin 2 (NPTX2) is reduced in cerebrospinal fluid (CSF) from patients with FTD, correlates with disease severity and with brain volume already at the presymptomatic stage in individuals with mutations in *C9orf72* or *GRN* [7, 8]. However, as a biomarker NPTX2 is challenged with the same problems as NEFL since reduced levels are also observed in other neurodegenerative diseases [9, 10].

In a previous study, we identified a set of fourteen proteins in CSF that could distinguish symptomatic mutation carriers (MC) from asymptomatic individuals with a high degree of accuracy [11]. Several of these proteins have also been found to be elevated in plasma from symptomatic MC [12], further strengthening their potential usefulness as biomarkers. However, information on how these biomarker candidates relate to important metrics such as brain atrophy or disease severity is still lacking. In the present study we have explored the association between these fourteen CSF biomarker candidates and symptom severity, as well as regional cortical and subcortical atrophy. The results indicate an association between many of the proteins and both symptom severity and brain atrophy, in some cases specifically for one of the genetic subgroups, further strengthening their potential as biomarkers for genetic FTD.

## Materials and methods

### Study cohort

The original cohort consisted of CSF samples from 221 participants, out of whom 202 had a viable 3T magnetic resonance imaging (MRI) scan from the same study-visit. These 202 individuals were selected for this follow-up study. All samples, images, and clinical, demographic, and genetic information were collected as a part of the Genetic Frontotemporal dementia initiative (GENFI, https://www.genfi.org/) according to standardized protocols [13]. During the study-visit, the participants underwent a standardized clinical assessment, CSF sampling and MR imaging. Genetic testing was done on pseudonymized samples, keeping participants and researchers blinded to the results. Sampling procedure and MRI acquisition protocols are thoroughly described elsewhere [13]. All participants provided written informed consent to their respective research sites.

### CSF protein measurements

CSF protein level measurements were obtained as a part of a previous study. For a full description of the methods used see Bergström et al. [11]. The proteins selected for the current study, based on their ability to separate symptomatic MC from controls, were neurofilament medium chain (NEFM), neuronal pentraxin 2 (NPTX2), VGF nerve growth factor inducible (VGF), aquaporin 4 (AQP4), apolipoprotein E (APOE), SEC63 homolog, protein translocation regulator (SEC63), apolipoprotein A1 (APOA1), protein tyrosine phosphatase receptor type N2 (PTPRN2), cathepsin S (CTSS), serpin family A member 3 (SERPINA3), complement 4 (C4), amphiphysin (AMPH), secreted phosphoprotein 1 (SPP1) and CD14 molecule (CD14).

### CDR

Symptom severity among the MC was estimated via the CDR plus NACC FTLD-NM scale (herein referred to as CDR) [14]. This is a modified version of the CDR plus NACC FTLD scale and includes assessments of neuropsychiatric and motor domains, capturing a larger part of the relevant phenotypic spectrum. For the statistical analysis the CDR plus NACC FTLD-NM sum-of-boxes score was used, which ranges from 0 to 27 points. CDR was only available for a subset of the study cohort: 52 C9-MC, 36 GRN-MC and 17 MAPT-MC. All analyses that included CDR were conducted in this subset.

### Imaging

The T1-weighted MRI images were processed through the TheHiveDB system using FreeSurfer 7.1.1 (http://surfer.nmr.mgh.harvard.edu/) [15, 16]. The results were visually inspected for quality control. Cortical thickness in 68 regions of interest (ROI), 34 in each hemisphere, based on the Desikan-Killiany atlas [17], along with 12 subcortical volumes (right and left hippocampus, amygdala, putamen, caudate, thalamus and pallidum), and CSF volume were extracted and used for the downstream analyses.

### Data pre-processing

Prior to statistical analysis all protein levels were log2-transformed, mean centered and unit variance scaled. The extracted ROI thicknesses/volumes were only centered and scaled. Next, the protein levels and ROIs were adjusted for confounders via residual-based adjustment [18]. Cortical thickness was adjusted for age, sex and collection site, subcortical volume was adjusted for age, sex, collection site and total intracranial volume, and the protein levels were adjusted for age, sex, collection site, and CSF volume. The effects of confounders were estimated in the NC using linear regression models with the confounders as predictors and regional cortical thickness, regional subcortical volume and protein levels as outcomes. Sum-to-zero contrasts were used for estimating the effect of the categorical variables sex and collection site. From these models, beta coefficients were extracted and used to adjust the ROI thicknesses/volumes and protein levels in the entire cohort through residual based normalization according to the formula below:

$$ Y_{{adj.}} = Y - \beta (X - \mu _{{ctrl}} ) $$

where $\:{Y}_{adj.}$ is the adjusted ROI thickness/volume or protein level, * Y* is the original ROI thickness/volumes or protein level, *X* is the design matrix for the specified confounders, excluding the intercept, $\mu _{{ctrl}}$ is the columns means of *X* in the NC subset, and $\:\beta\:$ is the confounder-associated coefficients estimated in the NC subset.

### Statistical analysis

Differences in age and age at onset between the genetic groups (C9-MC, GRN-MC and MAPT-MC) and NC, as well as differences in CDR between the genetic groups were calculated using the Kruskal-Wallis rank sum test. Differences in sex distribution between the genetic groups and NC were calculated using Pearson’s chi-squared test. The associations between protein levels and regional cortical thickness and subcortical volumes in the MC were estimated via linear regression models with ROI thicknesses/volumes as outcomes and protein levels as predictors. The associations between protein levels and CDR in the MC were similarly estimated via linear regression models with CDR sum-of-boxes score as outcome, protein level as predictor, and years of education included as a covariate. All p-values were adjusted for multiple testing using the Benjamini-Hochberg method for controlling false discovery rates. Protein – ROI association estimates were adjusted per ROI (*n* = 80), and protein – CDR estimates were adjusted per protein (*n* = 14). Data processing and statistical analysis was done using R (version 4.4.2) and the brain maps were made using the ggseg package [19].

## Results

The cohort included in this study consisted of 202 individuals, where 132 were mutation carriers (MC) and 70 were non-carrier controls (NC), with 38 MC having reached symptom onset (Table 1). Among the MC, 63 had repeat expansions in *C9orf72* (C9-MC), 46 had mutations in *GRN* (GRN-MC), and 23 had mutations in *MAPT* (MAPT-MC). There were no significant differences in age (*p* = 0.11), age at onset (*p* = 0.23) or sex distribution (*p* = 0.93) between any of the genetic groups and NC, nor were there any differences in CDR between the genetic groups (*p* = 0.34).

**Table 1:** Demographic overview of the cohort

	C9orf72	GRN	MAPT	Overall	*p*-value
	(*N* = 63)	(*N* = 46)	(*N* = 23)	(*N* = 202)	
Sex					
F	33 (52.4%)	26 (56.5%)	14 (60.9%)	112 (55.4%)	0.93
M	30 (47.6%)	20 (43.5%)	9 (39.1%)	90 (44.6%)	
Age					
Mean (SD)	49.7 (14.3)	52.2 (13.5)	46.8 (10.6)	49.0 (13.3)	0.11
Age at Onset					
Mean (SD)	56.9 (8.11)	59.9 (7.23)	52.4 (6.45)	57.0 (7.84)	0.23
Symptom status					
PMC	41 (65.1%)	37 (80.4%)	16 (69.6%)	94 (46.5%)	
SMC	22 (34.9%)	9 (19.6%)	7 (30.4%)	38 (18.8%)	
CDR-SoB					
Mean (SD)	2.90 (4.93)	3.13 (6.05)	2.15 (3.13)	2.05 (4.38)	0.34

PMC: presymptomatic mutation carrier, SMC: symptomatic mutation carrier, CDR-SoB: clinical dementia rating sum-of-boxes

We first estimated the associations between protein levels and disease severity, measured by CDR in all MC (Table 1). Five out of the fourteen proteins showed a significant association to CDR and the protein with the strongest association was NEFM (*p* < 0.001, β = 1.852), followed by PTPRN2 (*p* < 0.001, β = -1.633) and APOA1 (*p* = 0.004, β = 1.236). When stratified by genetic group two proteins were significantly associated with CDR in GRN-MC: PTPRN2 (*p* = 0.006, β = -2.228) and CD14 (*p* = 0.021, β = -2.769) (Table 2). In the MAPT-MC nine proteins had a significant association to CDR, with C4 having the strongest association (*p* < 0.001, β = 2.814), followed by SPP1 (*p* < 0.001, β = 2.547) and CTSS (*p* = 0.002, β = 2.179). In C9-MC none of the proteins showed significant associations to CDR after adjusting for multiple testing.

**Table 2:** P-values and beta-values from linear regression models estimating the association between protein biomarker candidates and CDR plus NACC FTLD-NM in all mutation carriers (MC) as well as the three genetic groups

	All mc		C9orf72		GRN		MAPT	
	*P*-value	β	*P*-value	β	*P*-value	β	*P*-value	β
NEFM	< 0.001*	1.852	0.063	1.746	0.182	1.519	0.018*	2.049
PTPRN2	< 0.001*	-1.633	0.092	-1.715	0.006*	-2.228	0.034*	1.76
APOA1	0.004*	1.236	0.092	1.403	0.148	1.988	0.246	1.044
VGF	0.004*	-1.184	0.223	-1.265	0.092	-1.744	0.501	0.603
SERPINA3	0.016*	1.123	0.092	1.63	0.910	0.269	0.002*	2.173
NPTX2	0.072	-0.771	0.561	-0.457	0.148	-2.1	0.714	0.274
SPP1	0.114	0.681	0.092	1.589	0.923	-0.074	< 0.001*	2.547
C4	0.120	0.704	0.206	1.472	0.802	-0.482	< 0.001*	2.814
AMPH	0.314	0.501	0.415	0.992	0.910	-0.236	0.003*	2.08
APOE4	0.420	0.375	0.449	0.633	0.625	0.937	0.178	1.322
AQP4	0.643	0.237	0.453	0.827	0.625	-0.728	0.011*	1.836
CD14	0.643	-0.243	0.415	0.97	0.021*	-2.769	0.002*	2.062
CTSS	0.671	0.206	0.453	0.884	0.625	-1.092	0.002*	2.179
SEC63	0.749	-0.109	0.836	0.138	0.910	-0.292	0.701	0.33

All p-values are adjusted for multiple testing. * indicates p-values below 0.05

**Fig. 1:**
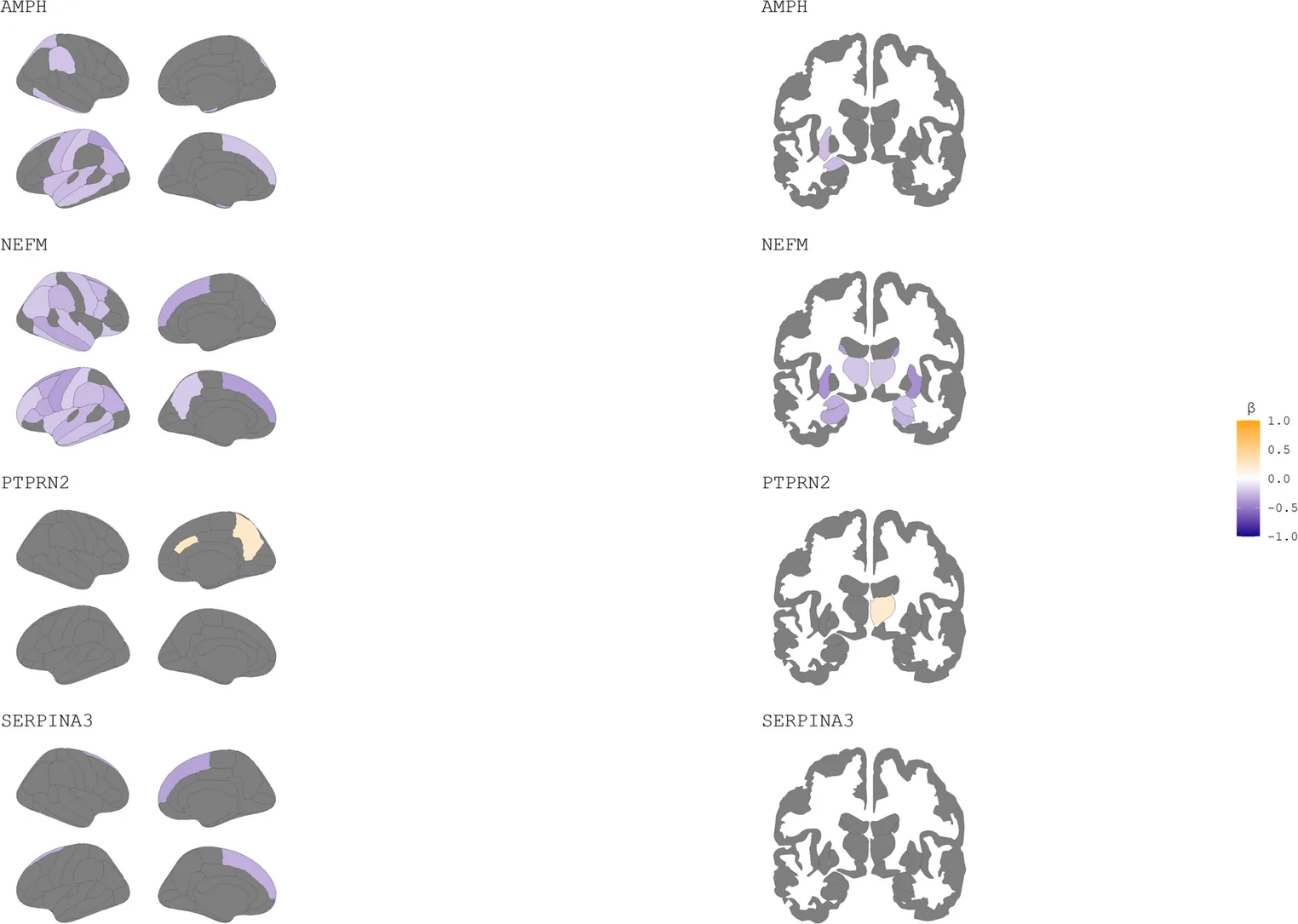
Brain maps showing associations between CSF protein levels and regional cortical thickness/subcortical volume. Color scale indicates the effect size ranging from 1 (orange) to -1 (dark purple). Only regions with adjusted p-values below 0.05 are colored (i.e. grey areas have adjusted p-values above the threshold)

Next, we tested the association between the levels of the CSF proteins and regional cortical thickness and subcortical volumes in all MC and observed significant associations for four proteins: NEFM, AMPH, PTPRN2 and SERPINA3 (Fig. 1). Elevated NEFM levels were associated with widespread cortical thinning affecting frontal, temporal and parietal regions, as well as reduced volume of the caudate, putamen, hippocampus, thalamus and amygdala. Elevated AMPH levels were associated with primarily left-sided atrophy of temporal, parietal and medial frontal regions, as well as the amygdala and putamen. Reduced levels of PTPRN2 were associated with right-sided atrophy of the precuneus, anterior cingulate cortex and the thalamus, while elevated levels of SERPINA3 were associated with bilateral atrophy of the superior frontal gyrus.

**Fig. 2:**
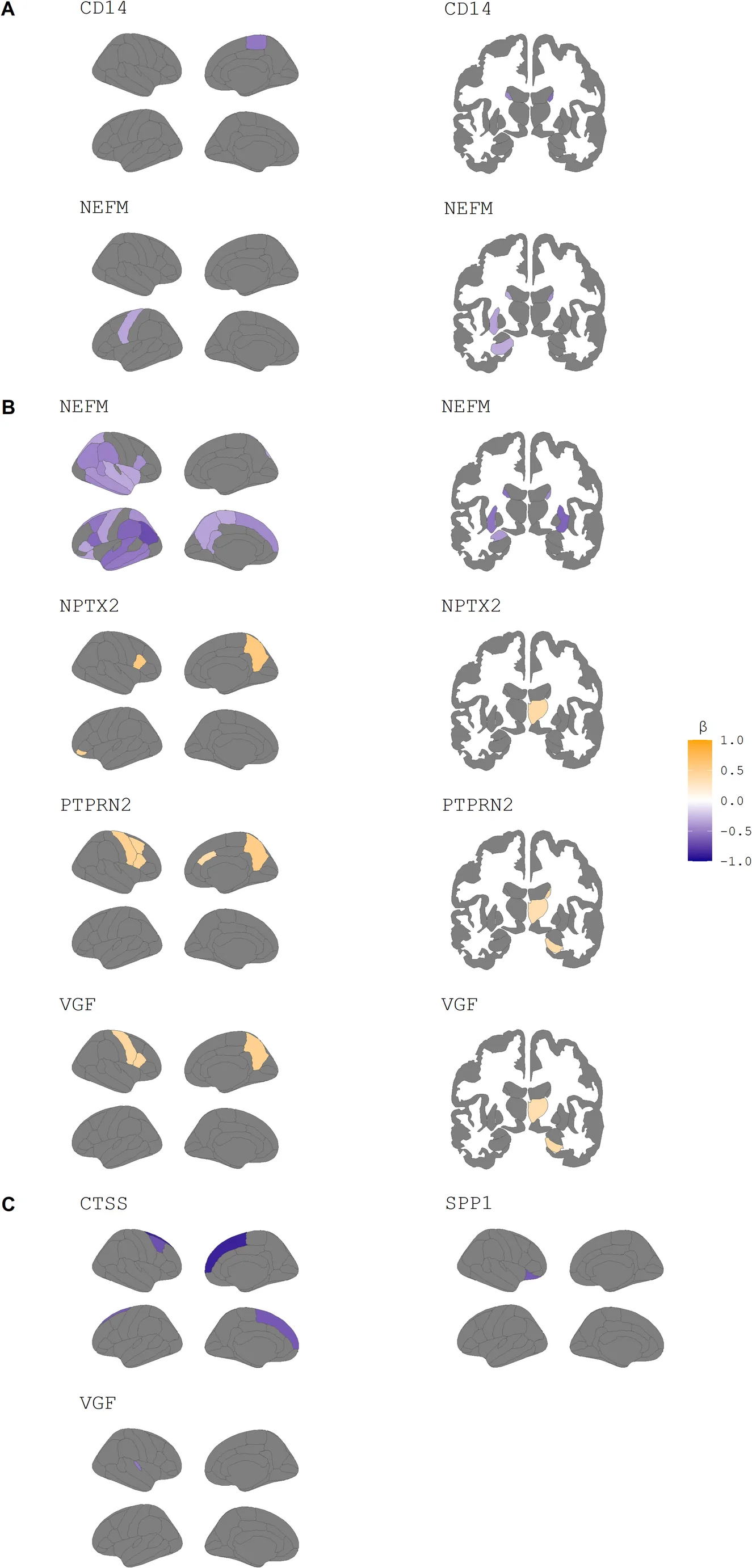
Brain maps showing associations between CSF protein levels and regional cortical thickness/subcortical volume in C9orf72 repeat expansion carriers (**A**), GRN mutation carriers (**B**) and MAPT mutation carriers (**C**). Color scale indicates the effect size ranging from 1 (orange) to -1 (dark purple). Only regions with adjusted p-values below 0.05 are colored (i.e. grey areas have adjusted p-values above the threshold)

Finally, we analyzed the association between protein levels and regional atrophy within the different genetic groups independently. In C9-MC, two proteins, CD14 and NEFM, were associated with cortical and subcortical atrophy (Fig. 2A). Elevated levels of CD14 were associated with right paracentral atrophy and bilateral atrophy of the caudate. In GRN-MC four proteins, NEFM, NPTX2, PTPRN2 and VGF, were associated with cortical and subcortical atrophy (Fig. 2B). Elevated NEFM levels were associated with widespread atrophy of frontal, temporal, and parietal regions, as well as the caudate, putamen and amygdala. Reduced levels of NPTX2, PTPRN2 and VGF were associated with atrophy of right-sided frontal and parietal regions, and of the right thalamus. PTPRN2 and VGF were additionally associated with atrophy of the right hippocampus, and PTPRN2 was associated with atrophy of the caudate. In MAPT-MC, three proteins, CTSS, SPP1 and VGF, were associated with cortical and subcortical atrophy (Fig. 2C). Elevated levels of CTSS were associated with bilateral atrophy of the superior frontal gyrus and of the right middle frontal gyrus. Elevated levels of SPP1 were associated with atrophy of the right lateral orbitofrontal cortex, while elevated levels of VGF were associated with atrophy of the right transverse temporal gyrus.

## Discussion

In this study we have shown how the CSF levels of fourteen previously identified protein biomarker candidates for genetic FTD are associated with symptom severity, cortical and subcortical atrophy in a cohort consisting of both symptomatic and presymptomatic mutation carriers, as well as non-carrier controls. Many of these biomarker candidates showed significant associations with symptom severity, either in one of the specific genetic groups or across all groups. Additionally, several of the CSF proteins were also associated with brain atrophy.

While five of the fourteen proteins included in this study had significant associations to CDR, only three of these proteins, NEFM, PTPRN2, and SERPINA3, were also associated with brain atrophy. The neurofilaments are well known markers of axonal damage and all three filaments (NEFL, NEFM, NEFH) are reported to be elevated in CSF from patients with FTD [5, 11, 20, 21]. Less is known about the roles of PTPRN2 and SERPINA3 in FTD. Both proteins are involved in innate immune response pathways, while PTPRN2 is additionally involved in vesicle-mediated secretion of neurotransmitters. Both synaptic dysfunction and neuroinflammation are known to play prominent roles in the pathogenesis of FTD, with synaptic dysfunction being an early event in the course of the disease and prominent neuroinflammation occurring around symptom onset [22–24]. Any therapeutical intervention targeting specific pathological processes, such as synaptic dysfunction or neuroinflammation, will require biomarkers that specific for these processes. Based on our results PTPRN2 and SERPINA3 could function as such biomarkers, although further validation is required.

The three genetic groups had distinct patterns of association between protein levels, atrophy and symptom severity. In C9-MC two proteins, CD14 and NEFM, were associated with cortical and subcortical atrophy, and none of the proteins had any significant association with CDR, although NEFM was just above the significance threshold. C9-MC usually experiences a slow decline in grey matter volume starting in early adulthood, while clinical symptoms progress more rapidly, with a relatively short disease duration, once onset has been reached [25–27]. It is possible that the discrepancy between the associations to symptom severity and atrophy reflects differences in the temporal dynamics of neurodegeneration and clinical symptom development, such that the slowly progressing structural and molecular changes are not linearly correlated with the rapid development of clinical symptoms. It is therefore possible that there exists a non-linear relationship between biomarker levels and symptom severity that we fail to capture in the current study.

In GRN-MC three proteins, NPTX2, PTPRN2 and VGF, were associated with a strikingly similar pattern of atrophy which included the right precuneus and inferior frontal gyrus, as well as the right thalamus and hippocampus. The precuneus and inferior frontal gyrus are involved in mentalization and theory of mind, while the thalamus and hippocampus are involved in, among other things, emotional blunting and positive emotion contagion [28, 29]. Loss of empathy and reduced responsiveness to others are hallmarks of behavioral variant FTD, together with loss of impulse control and behavioral disinhibition [30]. With their association to this distinct pattern of atrophy, NPTX2, PTPRN2 and VGF, have the potential to serve as biomarkers for specific symptoms of FTD, as well as their underlying cause. Such biomarkers would be valuable not only for diagnostic purposes, but also for future studies into the neurobiological substrates of specific clinical symptoms in FTD. In this study we only used the CDR sum-of-boxes as an overall estimate of symptom severity, without analyzing the association between protein levels and specific behavioral and functional domains. Future, more tailored, studies of these proteins would likely benefit from a more granular approach focusing on domain‑specific clinical features.

The group with the largest number of significant associations between protein levels and symptom severity was MAPT-MC. Out of the nine proteins with significant associations, five were specific to this genetic group. Two of these proteins, CTSS and SPP1, were additionally associated with cortical atrophy specifically in the MAPT-MC. Most of the proteins, six out of nine, are associated with neuroinflammation. A recent study of the CSF proteome in genetic FTD similarly highlighted the importance of altered immune function in MAPT-MC where 63 proteins, primarily involved in pathways linked to immune function, where upregulated in symptomatic MAPT-MC [31]. Similarly, a study of inflammatory plasma proteins in genetic FTD found elevated levels in symptomatic MAPT-MC compared to controls [32]. Together, this indicates that management of neuroinflammation can be a valid approach for the development of new disease-modifying treatments for this particular form of genetic FTD.

The main limitation of the study was the small sample size which, in combination with the heterogeneity of the disease, likely affected our ability to identify significant associations between CSF protein levels and brain atrophy or symptom severity. It also prevented certain subgroup analyses, such as stratification by both genetic group and symptom status. The cross-sectional design further prevented us from studying the association between protein levels and atrophy over time, as well as conversion from the presymptomatic stage to the symptomatic stage. Future longitudinal studies in larger cohorts will be necessary to fully understand the relationship between these biomarker candidates and genetic FTD.

In conclusion, in this study we have shown that several of the proteins included in this study are associated with clinically relevant pathological changes in genetic FTD. We therefore believe that they merit further investigation, particularly since there currently are so few viable biomarkers for this complex, heterogeneous disease.

## Supplementary Information

Below is the link to the electronic supplementary material.

Supplementary Material 1

## Data Availability

Anonymized data can be shared upon reasonable request from a qualified academic investigator for the purpose of replication of the results and procedures detailed in this article. All requests must agree with EU legislation on general data protection and must be in line with the decisions from the Ethical Review Board of Sweden. Data sharing must be regulated in a material transfer agreement and/or data processing agreement as appropriate.
