# Supplementary material for: CSF protein biomarkers are associated with atrophy and symptom severity in genetic FTD: a GENFI study
Source: J Neural Transm (Vienna). 2026 Feb 19;133(9):2339–47. doi: 10.1007/s00702-026-03118-y (PMC13687289; doi:10.1007/s00702-026-03118-y)
Supplement: Supplementary file 1 [file 702_2026_3118_MOESM1_ESM.docx]

# Supplementary materials

### The GENFI consortium author list

| **Author** | **Affiliation** |
| --- | --- |
| Rhian Convery | Department of Neurodegenerative Disease, Dementia Research Centre, UCL Queen Square Institute of Neurology, London, UK |
| Martina Bocchetta | Department of Neurodegenerative Disease, Dementia Research Centre, UCL Queen Square Institute of Neurology, London, UK |
| David Cash | Department of Neurodegenerative Disease, Dementia Research Centre, UCL Queen Square Institute of Neurology, London, UK |
| Sophie Goldsmith | Department of Neurodegenerative Disease, Dementia Research Centre, UCL Queen Square Institute of Neurology, London, UK |
| Kiran Samra | Department of Neurodegenerative Disease, Dementia Research Centre, UCL Queen Square Institute of Neurology, London, UK |
| David L. Thomas | Neuroimaging Analysis Centre, Department of Brain Repair and Rehabilitation, UCL Institute of Neurology, Queen Square, London, UK |
| Antonella Alberici | Centre for Neurodegenerative Disorders, Department of Clinical and Experimental Sciences, University of Brescia, Brescia, Italy |
| Enrico Premi | Stroke Unit, ASST Brescia Hospital, Brescia, Italy |
| Roberto Gasparotti | Neuroradiology Unit, University of Brescia, Brescia, Italy |
| Emanuele Buratti | ICGEB Trieste, Italy |
| Valentina Cantoni | Centre for Neurodegenerative Disorders, Department of Clinical and Experimental Sciences, University of Brescia, Brescia, Italy |
| Andrea Arighi | Fondazione IRCCS Ca’ Granda Ospedale Maggiore Policlinico, Neurodegenerative Diseases Unit, Milan, Italy |
| Vittoria Borracci | Fondazione IRCCS Ca’ Granda Ospedale Maggiore Policlinico, Neurodegenerative Diseases Unit, Milan, Italy |
| Maria Serpente | Fondazione IRCCS Ca’ Granda Ospedale Maggiore Policlinico, Neurodegenerative Diseases Unit, Milan, Italy |
| Tiziana Carandini | Fondazione IRCCS Ca’ Granda Ospedale Maggiore Policlinico, Neurodegenerative Diseases Unit, Milan, Italy |
| Emanuela Rotondo | Fondazione IRCCS Ca’ Granda Ospedale Maggiore Policlinico, Neurodegenerative Diseases Unit, Milan, Italy |
| Chiara Fenoglio | Dept. of Biomedical, Surgical and Dental Sciences, University of Milan, Milan, Italy; Fondazione IRCCS Ca’ Granda Ospedale Maggiore Policlinico, Neurodegenerative Diseases Unit, Milan, Italy |
| David Tang-Wai | The University Health Network, Krembil Research Institute, Toronto, Canada |
| Ekaterina Rogaeva | Tanz Centre for Research in Neurodegenerative Diseases, University of Toronto, Toronto, Canada |
| Miguel Castelo-Branco | Faculty of Medicine, ICNAS, CIBIT, University of Coimbra, Coimbra, Portugal. |
| Morris Freedman | Baycrest Health Sciences, Rotman Research Institute, University of Toronto, Toronto, Canada |
| Ron Keren | The University Health Network, Toronto Rehabilitation Institute, Toronto, Canada |
| Sandra Black | Sunnybrook Health Sciences Centre, Sunnybrook Research Institute, University of Toronto, Toronto, Canada |
| Sara Mitchell | Sunnybrook Health Sciences Centre, Sunnybrook Research Institute, University of Toronto, Toronto, Canada |
| Christen Shoesmith | Department of Clinical Neurological Sciences, University of Western Ontario, London, Ontario, Canada |
| Robart Bartha | Department of Medical Biophysics, The University of Western Ontario, London, Ontario, Canada; Centre for Functional and Metabolic Mapping, Robarts Research Institute, The University of Western Ontario, London, Ontario, Canada |
| Rosa Rademakers | Center for Molecular Neurology, University of Antwerp |
| Jackie Poos | Department of Neurology, Erasmus Medical Center, Rotterdam, Netherlands |
| Janne M. Papma | Department of Neurology, Erasmus Medical Center, Rotterdam, Netherlands |
| Lucia Giannini | Department of Neurology, Erasmus Medical Center, Rotterdam, Netherlands |
| Liset de Boer | Department of Neurology, Erasmus Medical Center, Rotterdam, Netherlands |
| Julie de Houwer | Department of Neurology, Erasmus Medical Center, Rotterdam, Netherlands |
| Rick van Minkelen | Department of Clinical Genetics, Erasmus Medical Center, Rotterdam, Netherlands |
| Yolande Pijnenburg | Amsterdam University Medical Centre, Amsterdam VUmc, Amsterdam, Netherlands |
| Mattias Nilsson | Department of Clinical Neuroscience, Karolinska Institutet, Stockholm, Sweden |
| Henrik Viklund | Karolinska University Hospital Huddinge |
| Vesna Jelic | Department of Neurobiology, Care Sciences and Society; Division of Clinical Geriatrics, Karolinska Institutet, Stockholm, Sweden; Cognitive clinic, Theme inflammation and Aging, Karolinska University Hospital, Solna, Sweden |
| Tobias Langheinrich | Division of Neuroscience and Experimental Psychology, Wolfson Molecular Imaging Centre, University of Manchester, Manchester, UK; Manchester Centre for Clinical Neurosciences, Department of Neurology, Salford Royal NHS Foundation Trust, Manchester, UK |
| Albert Lladó | Alzheimer’s disease and Other Cognitive Disorders Unit, Neurology Service, Hospital Clínic, Barcelona, Spain |
| Anna Antonell | Alzheimer’s disease and Other Cognitive Disorders Unit, Neurology Service, Hospital Clínic, Barcelona, Spain |
| Jaume Olives | Alzheimer’s disease and Other Cognitive Disorders Unit, Neurology Service, Hospital Clínic, Barcelona, Spain |
| Mircea Balasa | Alzheimer’s disease and Other Cognitive Disorders Unit, Neurology Service, Hospital Clínic, Barcelona, Spain |
| Nuria Bargalló | Imaging Diagnostic Center, Hospital Clínic, Barcelona, Spain |
| Sergi Borrego-Ecija | Alzheimer’s disease and Other Cognitive Disorders Unit, Neurology Service, Hospital Clínic, Barcelona, Spain |
| Alazne Gabilondo | Cognitive Disorders Unit, Department of Neurology, Donostia University Hospital, San Sebastian, Gipuzkoa, Spain; Neuroscience Area, Biodonostia Health Research Insitute, San Sebastian, Gipuzkoa, Spain |
| Ioana Croitoru | Neuroscience Area, Biodonostia Health Research Insitute, San Sebastian, Gipuzkoa, Spain |
| Mikel Tainta | Neuroscience Area, Biodonostia Health Research Insitute, San Sebastian, Gipuzkoa, Spain |
| Myriam Barandiaran | Cognitive Disorders Unit, Department of Neurology, Donostia University Hospital, San Sebastian, Gipuzkoa, Spain; Neuroscience Area, Biodonostia Health Research Insitute, San Sebastian, Gipuzkoa, Spain |
| Patricia Alves | Neuroscience Area, Biodonostia Health Research Insitute, San Sebastian, Gipuzkoa, Spain; Department of Educational Psychology and Psychobiology, Faculty of Education, International University of La Rioja, Logroño, Spain |
| Benjamin Bender | Department of Diagnostic and Interventional Neuroradiology, University of Tübingen, Tübingen, Germany |
| David Mengel | Department of Neurodegenerative Diseases, Hertie-Institute for Clinical Brain Research and Center of Neurology, University of Tübingen, Tübingen, Germany; Center for Neurodegenerative Diseases (DZNE), Tübingen, Germany |
| Lisa Graf | Department of Neurodegenerative Diseases, Hertie-Institute for Clinical Brain Research and Center of Neurology, University of Tübingen, Tübingen, Germany |
| Annick Vogels | Department of Human Genetics, KU Leuven, Leuven, Belgium |
| Mathieu Vandenbulcke | Geriatric Psychiatry Service, University Hospitals Leuven, Belgium; Neuropsychiatry, Department of Neurosciences, KU Leuven, Leuven, Belgium |
| Philip Van Damme | Neurology Service, University Hospitals Leuven, Belgium; Laboratory for Neurobiology, VIB-KU Leuven Centre for Brain Research, Leuven, Belgium |
| Koen Poesen | Laboratory for Molecular Neurobiomarker Research, KU Leuven, Leuven, Belgium |
| Pedro Rosa-Neto | Translational Neuroimaging Laboratory, McGill Centre for Studies in Aging, McGill University, Montreal, Québec, Canada |
| Maxime Montembault | Douglas Research Centre, Department of Psychiatry, McGill University, Montreal, Québec, Canada |
